# Polyurethane-based nanofibrous mat containing porphyrin with photosensitivity and bactericidal properties can promote cutaneous tissue healing in rats

**DOI:** 10.1186/s12951-023-02082-z

**Published:** 2023-09-04

**Authors:** Solmaz Saghebasl, Hassan Amini, Abbas Nobakht, Sanya Haiaty, Hesam Saghaei Bagheri, Parisa Hasanpour, Morteza Milani, Sepideh Saghati, Ozra Naturi, Mehrdad Farhadi, Reza Rahbarghazi

**Affiliations:** 1 Department of Medical Nanotechnology, Faculty of Advanced Medical Sciences, Tabriz University of Medical Sciences, Tabriz, Iran; 2 Drug Applied Research Center, Tabriz University of Medical Sciences, Tabriz, Iran; 3 Student Research Committee, Tabriz University of Medical Sciences, Tabriz, Iran; 4 Department of General and Vascular Surgery, Faculty of Medicine, Tabriz University of Medical Sciences, Tabriz, Iran; 5 Research Center of Biosciences & Biotechnology (RCBB), University of Tabriz, Tabriz, Iran; 6 Infectious and Tropical Diseases Research Center, Tabriz University of Medical Sciences, Tabriz, Iran; 7 Stem Cell Research Center, Tabriz University of Medical Sciences, Tabriz, Iran; 8 Department of Clinical Biochemistry and Laboratory Medicine, School of Medicine, Tabriz University of Medical Sciences, Tabriz, Iran; 9 Department of Tissue Engineering, Faculty of Advanced Medical Sciences, Tabriz University of Medical Sciences, Tabriz, Iran; 10 Department of Organic and Biochemistry, Faculty of Chemistry, University of Tabriz, Tabriz, Iran; 11 Department of Anatomical and Clinical Pathology, Tabriz University of Medical Sciences, Tabriz, Iran; 12 Department of Applied Cell Sciences, Faculty of Advanced Medical Sciences, Tabriz University of Medical Sciences, Tabriz, Iran

**Keywords:** Wound healing, Antimicrobial photodynamic therapy, Chitosan, Polyurethane, Cationic porphyrins, Electrospinning

## Abstract

The regeneration of cutaneous tissue is one of the most challenging issues in human regenerative medicine. To date, several studies have been done to promote cutaneous tissue healing with minimum side effects. The healing potential of polyurethane (PU)/Poly (caprolactone)–poly (ethylene glycol)–poly (caprolactone) (PCEC)/chitosan (CS) (PCS) nanofibrous mat with cationic photosensitizer meso tetrakis (N-methyl pyridinium-4-yl) porphyrin tetratosylate salt (TMP) was examined. The CS tripolyphosphate nanoparticles (CSNPs) were prepared and loaded by TMP to provide an efficient drug release system (TMPNPs) for delivery of TMP to promote wound healing. In in vitro setting, parameters such as bactericidal effects, cytocompatibility, and hemolytic effects were examined. The healing potential of prepared nanofibrous mats was investigated in a rat model of full-thickness cutaneous injury. PCS/TMP/TMPNPs nanofibers can efficiently release porphyrin in the aqueous phase. The addition of TMPNPs and CS to the PU backbone increased the hydrophilicity, degradation, and reduced mechanical properties. The culture of human fetal foreskin fibroblasts (HFFF2) on PCS/TMP/TMPNPs scaffold led to an increased survival rate and morphological adaptation analyzed by MTT and SEM images. Irradiation with a red laser (635 nm, 3 J/cm^2^) for the 30 s reduced viability of S. *aureus* and *E. Coli* bacteria plated on PCS/TMP and PCS/TMP/TMPNPs nanofibrous mats compared to PU/PCEC (PC) and PU/PCEC/CS (PCS) groups, indicating prominent antibacterial effects of PCS/TMP and PCS/TMP/TMPNPs nanofibrous (p < 0.05). Data indicated that PCS/TMP/TMPNPs mat enhanced healing of the full-thickness excisional wound in a rat model by the reduction of inflammatory response and fibrotic changes compared to the PC, and PCS groups (p < 0.05). Immunofluorescence imaging indicated that levels of Desmoglein were increased in rats that received PCS/TMP/TMPNPs compared to the other groups. It is found that a PU-based nanofibrous mat is an appropriate scaffold to accelerate the healing of injured skin.

**Graphical abstract:**

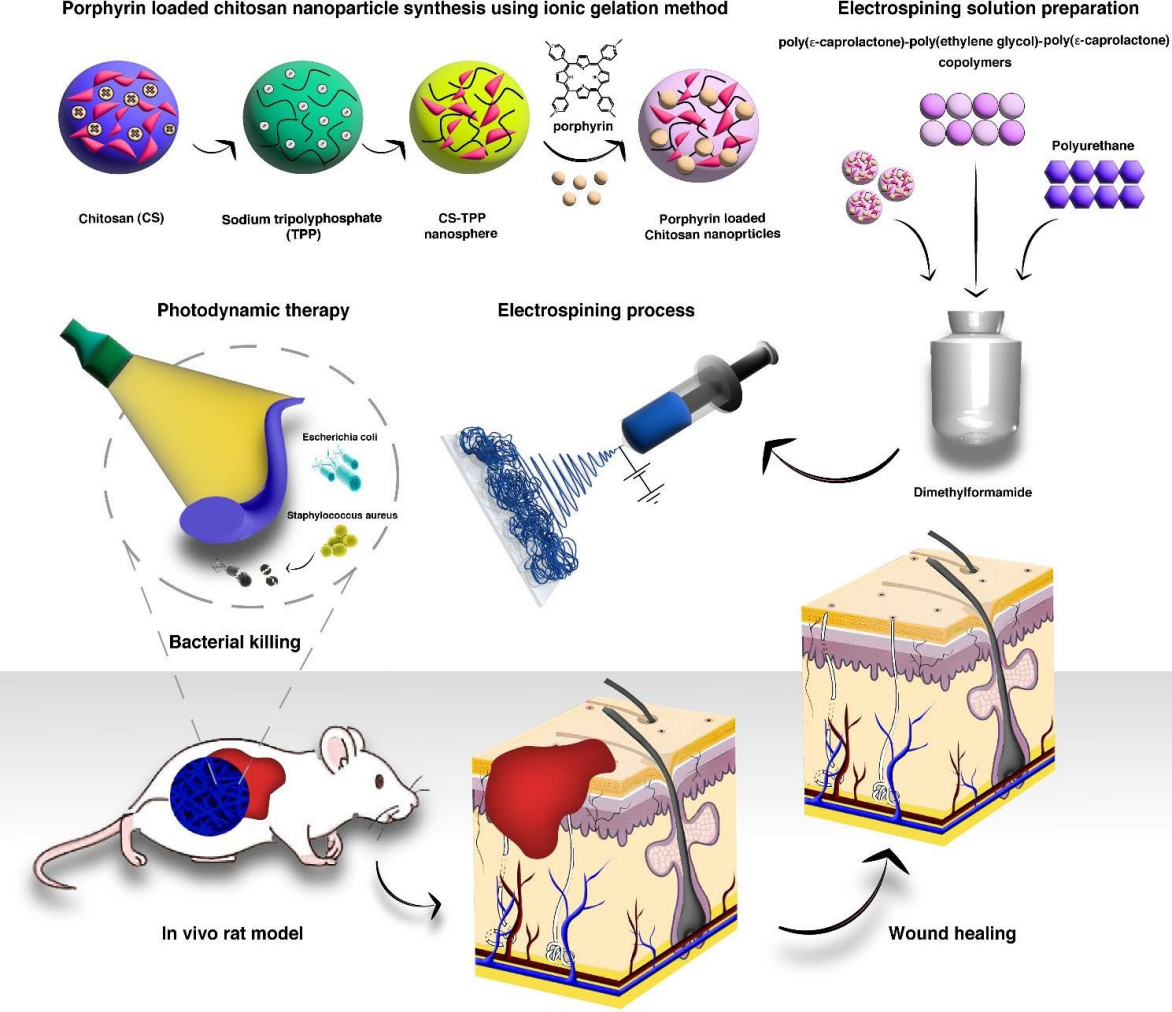

## Introduction

Skin with effective barrier properties prevents the invasion of microorganisms and pathogens into the deep layers [1]. Besides, the existence of external stratum corneum reduces transepidermal water loss and systemic dehydration [2]. It was suggested that common ulcers can be easily regenerated due to self-regeneration capacity in the skin while severe and extensive lesions are associated with compromised regeneration [3]. Of note, control, and management of inflammatory reactions and microbiota seem critical [4]. In recent years, multidisciplinary approaches such as tissue engineering modalities have been used along with traditional therapeutic regimens to expedite cutaneous tissue regeneration after damage [5]. The nanofibrous mats containing bioactive compounds are used as wound dressings in injured areas to accelerate the healing procedure meanwhile they can increase the possibility of bacterial infections [6]. Therefore, the development and fabrication of novel platforms with antibacterial properties are mandatory in restoring the function of injured cutaneous tissue.

Antimicrobial photodynamic therapy (APDT) is an alternative strategy in biomedical engineering with less invasive and less toxic effects to control skin microbiota [7]. In APDT-based modalities, the light-sensitive agents are regularly irradiated to generate singlet oxygen and free radicals [8]. The local accumulation of reactive oxygen species (ROS) exerts bactericidal effects on pathogenic microbes and prevents infectious dermatitis after the transplantation of engineered scaffolds [9]. Among several photosensitizer compounds, porphyrin and their compounds exhibit appropriate functionality in the generation of ROS [10]. Owing to its unique structure and excellent photophysical properties, the application of porphyrin derivatives has been pursued in biomedical fields [11]. It has been demonstrated that irradiation of cationic porphyrins effectively generates considerable amounts of ROS with significant bactericidal properties [12]. Meso tetrakis (N-methyl pyridinium-4-yl) porphyrin (TMP) with singlet oxygen quantum yield 0.74 in water solvent and high water solubility is one of the most important studied photosensitizers in APDT [13].

To provide an optimized electrospun platform mimicking the biophysical and mechanical properties of the native skin tissue, a structurally elastomeric nanofibrous network needs to be designed. Electrospun polyurethane nanofibers with excellent physical and biological properties such as unique elasticity, tunable mechanical properties, biocompatibility, degradability, good barrier properties, and also oxygen permeability are chosen as attractive candidates for wound dressing applications [14]. Recently, triblock Poly (caprolactone)–poly (ethylene glycol)–poly (caprolactone) (PCEC), a synthetic copolymer as a biomaterial has been extensively used in the field of tissue engineering due to its biocompatibility, biodegradability, and non-toxicity [15]. Both PCL and PEG have indicated acceptable results in forming a cell-scaffold complex. The desirable mechanical strength of PCL-based nanofibers makes these scaffolds extensively used in biomedical applications [16].

Nanofibrous-based implantable drug-eluting systems are commonly used for prolonged and sustained drug release kinetics in cutaneous tissue engineering [17]. To date, chitosan (CS) nanoparticles (NPs) have been widely used as an innovative drug delivery system to increase the stability of the drugs [18]. Favorable physicochemical properties like suitable biocompatibility, high-rate loading capacity, and low immunogenicity make CS a valid compound in the fabrication of delivery systems [19]. CS contains several hydroxyl functional groups and active amino groups with chelating and film-forming properties [6]. Despite the unique physicochemical properties, electrospinning of CS faces several challenges because of its poor solubility in common organic solvents. Besides, CS nanofibrous mats lack suitable mechanical properties, stability, and antibacterial activity in aqueous phases [20]. The blending of CS with other biocompatible polymers can, in part but not completely, circumvent these limitations [21].

In this work, biocompatible polyurethane (PU)-based nanofibrous mats have been fabricated with the purpose of cutaneous tissue wound healing and simultaneous bactericidal effects. The dressings were produced by simple electrospinning of PU/ PCEC/CS (PCS) co-loaded with free cationic photosensitizer meso tetrakis (N-methyl pyridinium-4-yl) porphyrin tetratosylate salt (TMP) and TMP-loaded CS tripolyphosphate NPs (TMPNPs) to acquire a novel efficient drug release system. In addition to physicochemical properties and cytocompatibility effects, the bactericidal properties of nanofibrous mats containing TMP and TMPNPs were examined on *Escherichia* and *Staphylococcus aureus* in in vitro conditions after exposure to 635 nm laser irradiation. The in vivo wound healing capacity and antibacterial effects were also monitored in a rat model of skin wound healing.

## Materials and methods

### Materials

PCL (Mn = 80,000), CS (75–85% deacetylated, medium molecular), sodium triphosphate pentabasic (TPP), poly 4, 4’-methylenebis(phenylisocyanate)-alt-14 butanediol/dipropyleneglycol/polycaprolactone, glutaraldehyde, meso tetrakis(N-methyl pyridinium-4-yl)porphyrin tetratosylate salt, 3,(4,5-dimethyithiazol-2-yl)2,5-diphenyl-tetrazolium bromide (MTT), N, N-dimethylformamide (DMF), Dimethyl sulfoxide (DMSO), and Stannous 2-ethyl hexanoate (Sn(Oct)_2_) were purchased from Sigma-Aldrich. Polyethylene glycol (PEG_4000_), Nutrient Broth, ε-caprolactone (ε-CL), and Acetic acid were obtained from Merck Chemical Co. Trypsin/EDTA solution (0.25%), Phosphate-buffered saline (PBS), Penicillin-Streptomycin (Pen-Strep), High-glucose content Dulbecco’s Modified Eagle Medium (DMEM/HG), and Fetal Bovine Serum (FBS) were supplied from GIBCO. Human fetal foreskin fibroblast (HFFF2) cells were obtained from the Iranian Cell Bank (Pasteur Institute, Iran). Gram-positive *Staphylococcus aureus* (ATCC 25,923) and Gram-negative *Escherichia Coli* (ATCC 25,922) were obtained from the National Collection of Industrial Microorganisms (NCIM, Iran).

### Synthesis and characterization of meso tetrakis (N-methyl pyridinium-4-yl) porphyrin-loaded Chitosan tripolyphosphate nanoparticles (TMPNPs)

TMP-encapsulated CSNPs were prepared using the ionic gelation technique with some modifications [22]. CS solution (5 g/L) was prepared by dissolving 7 g of CS powder in 1400 mL of acetic acid solution. Polyanion TPP solution (10 g/L) containing 4 mg TMP was also prepared. In the next step, TPP solution containing TMP was added drop by drop to the CS solution and homogenized under constant stirring at RT for 3 h. The mixture was centrifuged and the formed NPs were rinsed several times with distilled water. Finally, the obtained NPs were frozen at -80˚C and lyophilized overnight. Pure CSNPs were prepared as the control group. A UV/Vis spectrophotometer (Model: V-730, JASCO Inc., USA) was applied to quantify the amount of TMP within the collected supernatant at an absorption maximum of 421 nm with a standard curve of TMP. Finally, the encapsulation efficiency (EE %) and Loading capacity (LC %) of TMP into the NPs were calculated as follows:

Eq. 1 $${\rm{Encapsulation\, efficiency }}\left( {{\rm{EE \% }}} \right) = \left( {{\rm{A - B}}} \right){\rm{/A }} \times 100$$

Eq. 2 $${\rm{Loading}}\,{\rm{capacity }}\left( {{\rm{LC \% }}} \right){\rm{ = }}\left( {{\rm{A - B}}} \right){\rm{/C \times 100}}$$

Where A refers to the total mass of loaded TMP initially, B is the mass of free TMP in the supernatant and C stands for the total mass of TMPNPs

The morphology and particle size of prepared NPs were studied using FE-SEM (1430VP, LEO Electron Microscopy LTD, Cambridge, UK). To measure the surface charge and the stability of TMPNPs, zeta potential analysis was applied using dynamic light scattering (Malvern Instruments Ltd) in water at 25˚C. Moreover, the FT-IR analysis (Shimadzu FT-IR spectrometer, Kyoto, Japan) was utilized to confirm the functional groups and the chemical structure of the prepared NPs.

### Synthesis of poly (caprolactone)–poly (ethylene glycol)–poly (caprolactone) triblock copolymer

The triblock PCEC copolymer was synthesized using ring-opening polymerization of ε-CL in the presence of PEG_4000_ and stannous octoate according to previously published data [15]. Briefly, ε-CL and PEG [1:1.4 (w/w)] and stannous octoate with a concentration of 1wt% of total monomers were mixed and heated at 130˚C for 8 h under an atmosphere with rigid stirring. Next, the resultant product was dissolved in distilled H_2_O, dialyzed for three days at 4˚C, and then lyophilized for 24 h.

### Synthesis and characterization of the material

Briefly, PU/PCEC (PC) mixture with a 2:1 w/w ratio was dissolved in DMF by agitating for 12 h to form a 12% solution. To prepare the PU/PCEC/CS (PCS) nanofibrous solution, a certain amount of CS powder (3 wt%) was dispersed into DMF using stirring for 1 h followed by 20 min of sonication (Bandelin Electronic GmbH, Germany) to achieve a uniform emulsion. Then a proper amount of PC solution was blended with the CS solution and PCS concentration in the nanofibrous solution was fixed at 12%. To prepare a PCS solution containing 0.2 wt % TMP relative to PC, TMP was dissolved in DMF and added into the PCS solution for at least 1 h before electrospinning. Electrospinning solutions containing TMP and TMPNPs were prepared in the same manner based on the TMP loading capacity in the TMPNPs and the maximum quantity of TMP in the PCS nanofibers. The prepared solutions were electrospun over a voltage range from 16 to 18 KV, 1ml/h flow rate, a distance of 20 cm with a drum rotation of 320 rpm at RT.

The chemical structure of prepared nanofibers was analyzed using FT-IR analysis (Shimadzu 8400 S, Kyoto, Japan) in the range of 4000 − 400 cm^− 1^ and resolution of 4 cm^− 1^ with KBr pellet at room temperature. The morphology and fiber diameter of the nanofibrous mats were determined using FE-SEM (1430 VP, LEO Election Microscopy Ltd, Cambridge, UK) after gold sputtering.

The rate of surface wettability was determined through a contact angle measuring instrument (PGX, Sweden). For this purpose, the prepared nanofibrous mats were cut into sizes of 1 × 1 cm^2^, and then a droplet of distilled water was dropped on the substrate surface at RT. The tensile properties of the dry nanofibrous mats were investigated using a tensile tester (Z010, Zwick/Roell) using a 10 N load at a cross-head speed of 10 mm/min at RT. The fibers in sizes of 50 × 10 mm^2^ were used for tensile characterization. To evaluate the in vitro degradation rate, fabricated nanofibrous mats were weighed (W_i_), placed in a tube containing 10 ml of DMEM/HG with 10% FBS and 1% Pen-Strep and then incubated at 37˚C for 30 days. At the predetermined intervals, the nanofibrous mats were collected, washed, vacuum-dried, and weighted (W_t_). Finally, the degradation rate was evaluated using Eq. (3).

Eq. 3 $${\rm{Degradation }}\left( {\rm{\% }} \right){\rm{ = }}\left( {{{\rm{W}}_{\rm{i}}}{\rm{ - }}{{\rm{W}}_{\rm{t}}}} \right){\rm{/}}{{\rm{W}}_{\rm{i}}}{\rm{ \times 100}}$$

The release of TMP from nanofibrous PCS/TMP and PCS/TMP/TMPNP mats was measured in in vitro conditions. For this purpose, prepared fibers (100 mg) were placed in a bottle containing 10 ml PBS inside a shaker at 37˚C. At predetermined time intervals, 3 ml of buffer solution was sampled and replaced with an identical volume of PBS. The TMP content in the PBS solution was estimated at λ_max_ = 421 nm by UV/Vis spectrophotometer (Shimadzu 2550) compared with the standard curve of TMP in the same media. All tests were performed in triplicate.

### In vitro bactericidal evaluation

To evaluate the bactericidal properties of nanofibrous mats containing TMP, bacterial viability was monitored using turbidity and MTT assays after being exposed to low-level laser irradiation. In short, both gram-positive *Staphylococcus aureus* (*S*. *aureus*) and gram-negative *Escherichia Coli* (*E*. *Coli*) were cultured in Nutrient Broth solution to achieve a concentration of 1.5 × 10^8^ CFU/ml. Before irradiation, UV-sterilized nanofibrous mats were placed at the bottom surface of 96-well plates and 100 µl broth medium was overlaid to each well and kept at RT for 30 min. The samples were exposed to irradiation with a wavelength of 632 nm at a power density of 3 J/cm^2^ for 30 s (Technika LTD, Serial No. 20044GT0222). Forty-eight hours after completion of the irradiation protocol, the turbidity of the incubation media, representing the bacterial growth was determined using a microplate reader (Dynex MRX) at 600 nm. The bactericidal properties of nanofibrous mats were measured as follows:

Eq. 4 $${\rm{Antibacterial}}\,{\rm{efficiency }}\left( {\rm{\% }} \right){\rm{ = 1 - O}}{{\rm{D}}_{\rm{2}}}{\rm{/O}}{{\rm{D}}_{\rm{1}}}{\rm{ \times 100}}$$

Where OD_2_ is the optical density of the bacteria in solutions of nanofibrous mats and OD_1_ stands for the optical density of the control group containing nanofibrous mat-free bacteria.

To calculate the viability of bacteria after being exposed to laser irradiation, an MTT assay was performed. Nutrient Broth was carefully discarded and100 µl MTT solution (2 mg/ml) was added to each well and incubated for 3–4 h at 37˚C. The procedure was continued with the MTT solution removal and addition of DMSO. After 20 min, the optical density was read at 590 nm by a microplate reader and the bactericidal properties were calculated according to Eq. (4). This assay was done in duplicate (each in 6).

### In vitro biocompatibility assays

#### Hemolysis assay

To evaluate the blood compatibility, nanofibrous mats were cut into 1 × 1 cm^2^ sheets and incubated with 1 ml of the rat freshly diluted citrated blood saline solution at 37˚C for 60 min in a shaking incubator. After that, samples were centrifuged at 8000 rpm for 3 min and the absorbance of each supernatant was read at 540 nm using a microplate reader. In the control groups, the normal saline and deionized water-treated RBCs were used as the negative and positive controls, respectively. The hemolysis percentage of nanofibrous mats was obtained according to Eq. (5):

Eq. 5 $$\text{H}\text{e}\text{m}\text{o}\text{l}\text{y}\text{s}\text{i}\text{s} \left(\text{\%}\right)=\frac{{\text{O}\text{D}}_{\text{S}\text{a}\text{m}\text{p}\text{l}\text{e}}-{\text{O}\text{D}}_{\text{N}\text{e}\text{g}\text{a}\text{t}\text{i}\text{v}\text{e} \text{c}\text{t}\text{r}}}{{\text{O}\text{D}}_{\text{P}\text{o}\text{s}\text{i}\text{t}\text{i}\text{v}\text{e} \text{c}\text{t}\text{r}}-{\text{O}\text{D}}_{\text{N}\text{e}\text{g}\text{a}\text{t}\text{i}\text{v}\text{e} \text{c}\text{t}\text{r}}}\times 100$$

#### Blood clotting index (BCI)

To measure the BCI value, mats were cut into 1 × 1 cm^2^ sheets and placed in the glass plates. Then, 100 µl of fresh rat blood was gradually added to the fibrous mats to cover them and incubated for 5 min at 37˚C. Using 10 ml ultra-pure water, nanofibrous mats were carefully washed. In the control group, 100 µl of pure blood plus 10 ml of ultra-pure water was used. After that, plates were placed in a shaker at 37˚C with 30 rpm for 10 min and then the absorbance was read at 540 nm with a microplate reader. BCI value was obtained according to Eq. (6):

Eq. 6 $$\text{B}\text{C}\text{I} \left(\text{\%}\right)=\frac{{OD}_{Sample}}{{OD}_{Control}}\times 100$$

#### Proliferation assay

The cytocompatibility properties of nanofibrous mats were assessed on human fetal foreskin fibroblasts (HFFF2 cell line) using an MTT assay. The nanofibrous mats were disinfected in 70% ethanol for 1 h and placed under ultraviolet light overnight inside a laminar flow hood. HFFF2 cells were cultured in DMEM/HG culture medium with 10% FBS and 1% Pen-Strep. At 70–80% confluence, cells were detached using 0.25% Trypsin-EDTA solution, washed with PBS three times. About 1 × 10^4^ cells were suspended in 200 µl DMEM/HG containing 10% FBS and 1% Pen-Strep and transferred onto each well of 96-well plates pre-coated with sterile nanofibrous mats. The cells were maintained under standard conditions (95% humidity, 5% CO_2,_ and 37˚C). The survival rate was determined after 24, 48, and 72 h using an MTT assay. All procedures were done under sterile conditions.

#### Cell adhesion assay

To check whether human HFFF2 cells can attach to the surface of nanofibrous mats, SEM images were taken. After 72 h, nanofibrous mats were washed with PBS for 10 min and fixed using glutaraldehyde (2.5% v/v) solution at 4˚C for 1 day. The next day, samples were dehydrated using the ascending concentrations of ethanol solutions (from 50 to 100%). Following gold sputtering, samples were imaged using SEM apparatus.

### In vivo studies

#### Animal issue

All in vivo assays were approved by the local ethical committee of Tabriz University of Medical Sciences (IR.TBZMED.AEC.1402.010) and procedures were conducted according to the guideline of The Care and Use of Laboratory Animals (NIH Publication No. 85 − 23, revised 1996). In this study, the regenerative properties of nanofibrous mats were analyzed in a rat model of skin wound healing. To this end, mature male Wistar rats (200 ± 15 g) were enrolled in this study. Animals were kept under standard conditions [12 h: 12 h light-dark cycle and temperature of 22 ± 2 °C] at the animal house of the Faculty of Advanced Medical Sciences with free access to tap water and chewing pellets.

#### Wound healing assay

In this study, thirty-six rats were randomly allocated into four groups (n = 9 per group) including mat-free, PC, PCS, and PCS/TMP/TMPNPs groups. The number of rats examined at each time was 3. To induce cutaneous surgical wounds, rats were anesthetized using a combination of ketamine (75 mg/kg) and xylazine (3 mg/kg). The dorsal fur was shaved and the surgical sites were disinfected using 70% EtOH and povidone-iodine solutions. After that, a circular full-thickness defect with a diameter of 20 mm was generated on each rat using forceps and surgical scissors. In the mat-free control rats, the incision areas were not covered using nanofibrous mats. In other groups, the nanofibrous mats were placed on the surface of the wound area. On days 7, 14, and 21, transplanted sites were photographed with an Apple iPhone XS Max from a distance of approximately 20 cm. Wound healing measurement was calculated directly on the wound using the longest length on the surface in any direction by a ruler. Finally, the wound closure rate (%) was determined using the following formula;

Eq. 7 $$\text{W}\text{o}\text{u}\text{n}\text{d}\, \text{c}\text{l}\text{o}\text{s}\text{u}\text{r}\text{e}\, \left(\text{\%}\right)\frac{\text{w}\text{o}\text{u}\text{n}\text{d}\, \text{a}\text{r}\text{e}\text{a}\, \text{a}\text{t}\, 0 \text{d}\text{a}\text{y}-\text{w}\text{o}\text{u}\text{n}\text{d}\, \text{a}\text{r}\text{e}\text{a}\, \text{a}\text{t} \text{n}\, \text{d}\text{a}\text{y}}{ \text{w}\text{o}\text{u}\text{n}\text{d}\, \text{a}\text{r}\text{e}\text{a}\, \text{a}\text{t}\, 0 \text{d}\text{a}\text{y}}$$

#### Histological analysis

After the completion of the experimental period, rats were euthanized using an overdose of Ketamine and Xylazine. Samples were taken at different time points (7, 14, and 21). For general histological analysis, samples were fixed in a 10% buffered-formalin solution for 24 h. 5 μm thick slides were prepared from paraffin-embedded cutaneous samples, stained with Hematoxylin-Eosin (H & E) solution, and photographed using an optical microscope (Labomed, USA). To monitor the re-epithelialization of transplanted areas, protein levels of Desmoglein were assessed using immunofluorescence (IF) analysis. To monitor Desmoglein levels, freshly collected samples were embedded in OCT and 5 μm thick slides were prepared using a cryostat system (Leica). Slides were washed with PBS for 10 min and permeabilized using the 0.1% Triton-X100 for 10 min. Following blocking with 1% BSA for 1 h, samples were incubated with an anti-Desmoglein antibody (Cat no. 901-419-022311) overnight at 4˚C. The procedure was continued with PBS washes and the addition of a Cy3-tagged (Cat No. E-AB-1011; Elabscience) secondary antibody. After PBS washes, nuclei were stained using DAPI (1 µg/ml; Sigma-Aldrich) for 40 s and slides were imaged using BX41 Olympus microscopy.

### Statistical analysis

Results were analyzed with Graph Pad Prism software (ver.8) using One-Way ANOVA followed by Turkey’s multiple comparisons tests. In this study, p < 0.05 was considered statistically significant.

## Results and discussion

### Characterization of chitosan tripolyphosphate nanoparticles

**Fig. 1:**
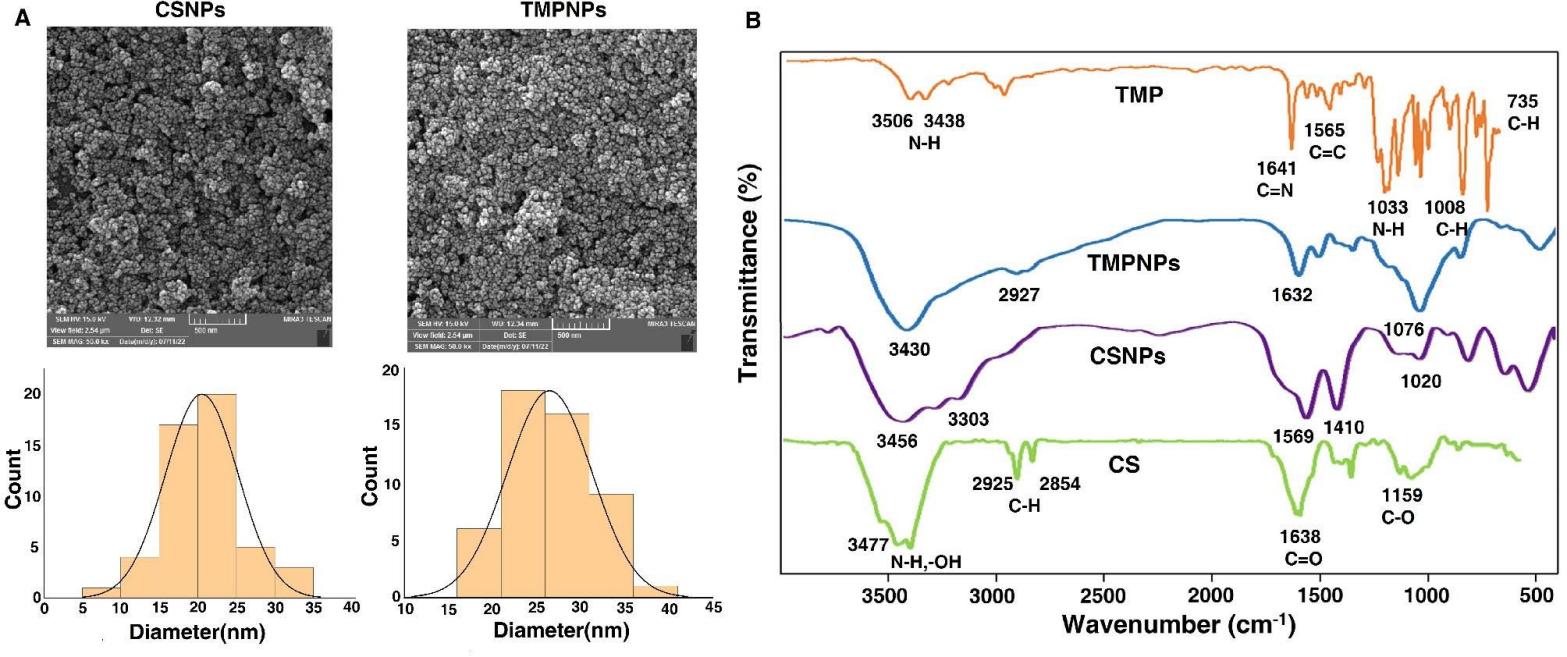
Monitoring physicochemical properties of the prepared NPs using FE-SEM (**A**) and FTIR (**B**) analyses. FE-SEM images revealed normal size distribution for prepared CSNPs, and TMP-loaded CSNPs (TMPNPs) (**A**). FT-IR spectra of CS, CSNPs, TMPNPs, and pure TMP (**B**). Data indicated appropriate incorporation of TMP into CS structure

In this study, CSNPs were synthesized after the addition of TMP to TPP and CS using the gelation procedure and encapsulation to maintain and stabilize photosensitizer TMP in aqueous media. The simultaneous loading of porphyrins with other nanostructured materials can lead to a self-quenching effect and hydrophobic π-π interactions of planar porphyrins [23]. It was suggested that these features can inhibit or decrease the property of porphyrins to generate reactive singlet molecular oxygen (^1^O_2_) [13]. Therefore, attempts should be directed toward the application of sophisticated synthesis protocol to preserve the ^1^O_2_ generation potential of porphyrins in the final structure to yield therapeutic outcomes for biomedical applications and phototherapy [24]. According to the SEM images (Fig. 1A), both CSNPs and TMP-loaded CSNPs (TMPNPs) exhibited spherical morphology with an average particle size of 20.51 ± 4.7 nm before TMP encapsulation. These values reached 26.50 ± 4.8 nm after TMP encapsulation. High encapsulation efficiency and high drug loading capacity are expected for an ideal and efficient drug delivery system [25]. In this study, UV/Vis spectroscopy and Eq. (1) and Eq. (2) were utilized to determine encapsulation efficiency and drug loading capacity. Using the TMP standard curve, the amount of TMP loaded into the CSNPs and its encapsulation efficiency was found to be 70.21 and 90.75% respectively. According to the DLS data, it confirmed that TMP was successfully loaded inside CSNPs. Based on obtained data, the average zeta potential of free CSNPs was − 10.9 mV due to the negative charges of TPP on the surface of NPs, whereas these values reached + 8.42 mV in TMPNPs after incorporation of TMP into the CSNP structure. These data indicate the successful encapsulation of TMP by the CSNPs. We also noted that neat TMP had a small positive zeta potential of approximately + 0.03 mV. Using FT-R spectroscopy, the loading of TMP into CSNPs was also assessed. Figure 1B shows the FT-IR spectra of CS, CSNPs, TMPNPs, and TMP. The FT-IR spectrum of TMP displayed peaks at 1566, 1033, 1008, 801, and 735 cm^-1^, indicating C = C stretching vibrations, N-H bending (in-plane), C-H rocking vibrations, C-H bending (out-of-plane), respectively. The strong band at 1641 cm^-1^ correlates with the stretching vibration of C = N in pyridyl rings [26]. The characteristic bonds of CS at 3477, 2925, 2854, and 1638 cm^-1^ are associated with N-H stretching vibrations, symmetric and asymmetric stretching of the methylene group, CH stretching, and carbonyl vibration [27]. As can be observed in Fig. 1B, the spectrum of TMPNPs exhibits bands of both CS and porphyrin with slight shifts, indicating the successful loading of TMP into the chitosan nanoparticles.

### Characterization of nanofibrous mats

**Fig. 2:**
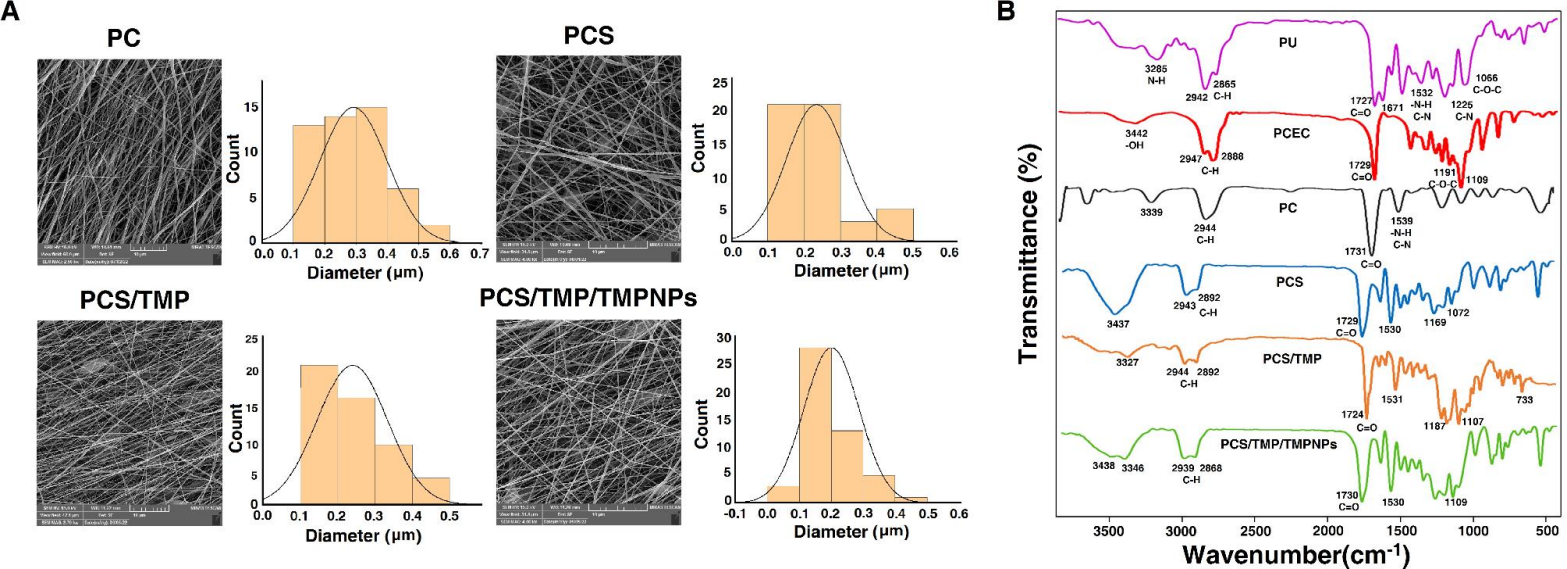
Monitoring physicochemical properties of the prepared scaffolds using FE-SEM (**A**) and FTIR (**B**) analyses. FE-SEM images and calculation indicated a relatively even distribution diameter size in the prepared nanofibrous mats (**A**). FT-IR spectra of pure PU, PCEC, PC, PCS, PCS/TMP, and PCS/TMP/TMPNPs nanofibrous mats (**B**). Data represents successful synthesis procedure in the preparation of PCS/TMP, and PCS/TMP/TMPNP nanofibrous mats

The surface morphology and ultrastructural properties of scaffolds play a significant role in modulating cellular adhesion, proliferation, and neotissue formation in the tissue engineering era [28]. According to previous data, the average fiber diameter and homogeneity of fibers can strongly affect the behavior of scaffolds and their interaction with cells [29]. As a common belief, smaller fiber diameters provide a favorable interaction of living cells with the scaffold, and it is suggested that the cell growth rate can reduce with increasing fiber diameter [30]. In Fig. 2A, The FE-SEM images of the designed mats along with their size distributions are displayed. All nanofibrous mats exhibited homogeneous and smooth surface morphology with bead-less constructions. Based on the SEM panel, the mean diameter of the PC nanofibers was 292 ± 107 nm, while those for the fabricated PCS, PCS/TMP, and PCS/TMP/TMPNPs mats were 239 ± 94, 234 ± 82, and 200 ± 86 nm, respectively. Upon the incorporation of CS into the PC nanofibrous mats, the mean diameter of fibers was decreased. According to the data, a minimum diameter size was notified in the PCS/TMP/TMPNPs group. Moreover, there is no evidence of TMP aggregation on the structure of nanofibers, indicating that PCS and TMP are miscible. The results indicated that there is no significant difference in fiber diameter and morphology when TMP was added to the PCS electrospinning solution.

FT-IR spectroscopy was utilized to verify the presence of PU, PCEC, CS, TMP, and TMPNPs in nanofibrous mats. In Fig. 2B, the FT-IR spectrum of pure PU is indicated at 1727 cm^− 1^ and 1671 cm^− 1^(C = O stretching), 1225 cm^− 1^ (coupled C-N and C-O stretching), and 1086 cm^− 1^ (C-O-C stretching). The vibrational peaks seen at 2942 cm^− 1^ and 2885 cm^− 1^ are due to CH stretch. Additionally, the PCEC triblock copolymer spectrum displays bands at 3442 cm^− 1^ (O-H stretching), 1730 cm^− 1^ (C = O stretching), and 1109–1249 cm^− 1^ (C-O-C stretching). We also noted that bands at 2888 cm^− 1^ and 2947 cm^− 1^ are related to C-H stretching vibrations. These features indicate the successful development of PC, PCS, PCS/TMP, and PCS/TMP/TMPNPs nanofibrous mats. As mentioned above, the characteristic absorption bands of the functional groups in pure samples are present in the structure of nanofibrous mats, indicating the perfect blending of samples in the fabricated nanofibrous mats.

**Fig. 3:**
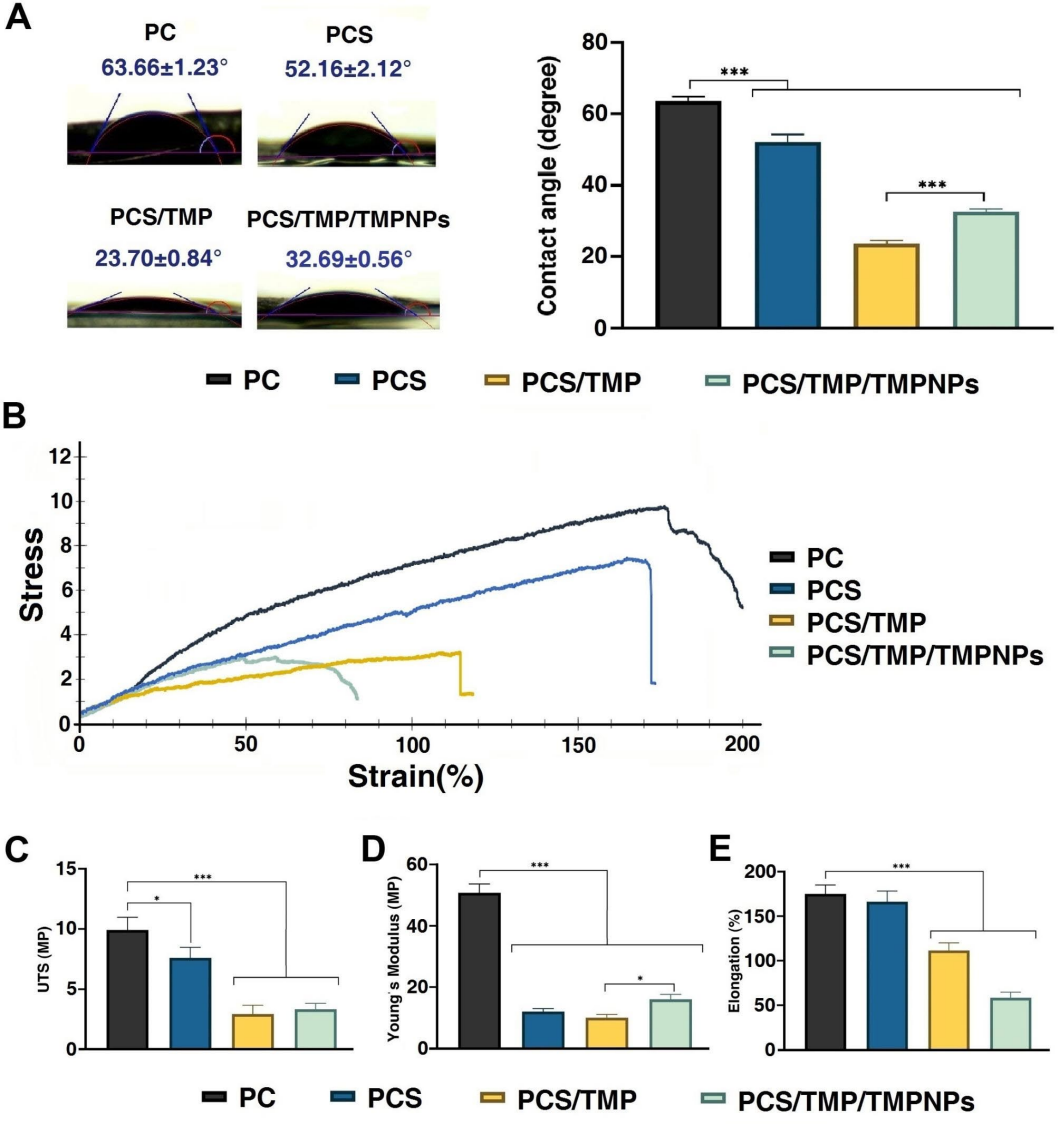
Measuring the wettability of nanofibrous mats using WCA (**A**). Data indicated the reduction of WCA values in groups PCS/TMP, and PCS/TMP/TMPNPs compared to other groups (**A**). Tensile stress-strain curves of nanofibrous mats (**B**). Ultimate tensile strength (UTS) (**C**), Young’s modulus (**D**), and Elongation at break values of nanofibers (**E**). One-Way ANOVA with Turkey post hoc analysis (n = 3; *p < 0.05 and ***p < 0.001)

It has been found that the surface wettability of engineering scaffolds is one of the key features required for restoring damaged skin [31]. To evaluate the surface wettability of fabricated mats, the water contact angle (WCA) has been determined. As shown in Fig. 3A, the WCA values for PC, PCS, PCS/TMP, and PCS/TMP/TMPNPs nanofibers were 63.66 ± 1.23, 52 ± 2.12, 23.70 ± 0.84, and 32.69 ± 0.56°, respectively, indicating their hydrophilic nature. Data indicate a significant reduction of WCA in PCS, PCS/TMP, and PCS/TMP/TMPNPs substrates compared to the PC group (*p*_PC_ vs. _PCs_ <0.001; *p*_PC_ vs. _PCS/TMP_ <0.001; *p*_PC_ vs. _PCS/TMP/TMPNPs_ <0.001). It seems that these effects were more prominent in PCS/TMP group compared to PCS/TMP/TMPNPs (*p*_PCS/TMP_ vs. _PCS/TMP/TMPNPs_<0.001). In this study, the observed small WCA values in the PCS/TMP nanofibrous mat could be associated with the presence of water-soluble cationic porphyrin (TMP) inside the nanofibrous structure. It is postulated that the integration of TMP induced a hydrophilic behavior for the nanofibrous PCS/TMP mats.

Along with the above-mentioned descriptions, scaffolds should exhibit favorable elasticity and mechanical strength to exert efficient healing properties by the regulation of cellular attachment, and proliferation [32]. Here, the mechanical behavior of the fabricated nanofibrous mats was evaluated through the tensile strength test, and the obtained results are presented in Fig. 3B. Data indicated that the ultimate tensile strength value of PCS mats was 7.61 ± 0.38 MPa and these properties were decreased by the incorporation of CS. It confirmed that PC nanofibers exhibited an ultimate tensile strength of about 9.92 ± 0.54 MPa (*p* < 0.05). The mechanical properties of the designed nanofibrous mats could be influenced by several factors including average fiber diameter, fiber composition, morphology, and homogeneity of fibers [33]. The possible effects of TMP and TMPNPs were also analyzed on the mechanical properties of final nanofibrous mats. Results revealed the lack of appropriate mechanical strength in the PCS/TMP group. This feature can be due to reduced interactions between polymer chains with the addition of the TMP. According to data presented in Fig. 3B, the tensile strength reached 3.34 ± 0.31 MPa in PCS/TMP/TMPNPs and 2.95 ± 0.23 MPa in PCS/TMP groups. Based on obtained results (Fig. 3C), there is an obvious difference in the UTS value of nanofibrous PC mats compared to the PCS/TMP and PCS/TMP/TMPNPs groups (*p*_PC_ vs. _PCs_ <0.05; *p*_PC_ vs. _PCS/TMP_ <0.001; *p*_PC_ vs. _PCS/TMP/TMPNPs_ <0.001). Commensurate with these descriptions, the fabricated nanofibrous mats are appropriate biomaterial dressings for wound healing applications. It is suggested that suitable tensile strength for a wound dressing is in the range of 0.7–18 MPa [34]. Moreover, the PC nanofiber exhibited Young’s modulus of 50 ± 3.65 MPa and the elongation at break 175 ± 4.12 MPa. Data indicated that the addition of CS into PC nanofibers reduced significantly Young’s modulus (12.42 ± 1.37 MPa; *p*_PC_ vs. _PCs_ <0.001) (Fig. 3D). A similar pattern was achieved in PCS/TMP and PCS/TMP/TMPNPs groups (*p*_PC_ vs. _PCS/TMP_ <0.001; *p*_PC_ vs. _PCS/TMP/TMPNPs_ <0.001). In this study, the elongation at break of PC was 166.26 ± 0.87 MPa (Fig. 3E). Besides, the incorporation of TMP to nanofibrous mats caused a reduction in Young’s modulus and elongation at break (p < 0.05). This decrease can be attributed to the poor physical properties of TMP. Furthermore, TMPNPs blending with PCS/TMP mats lead to increased Young’s modulus (16.32 ± 1.94 MPa; *p*_PCS/TMP_ vs. _PCS/TMP/TMPNPs_<0.05), whereas the elongation at break significantly decreased to 58.47 ± 2.12 MPa (*p*_PCS/TMP_ vs. _PCS/TMP/TMPNPs_<0.001).

**Fig. 4:**
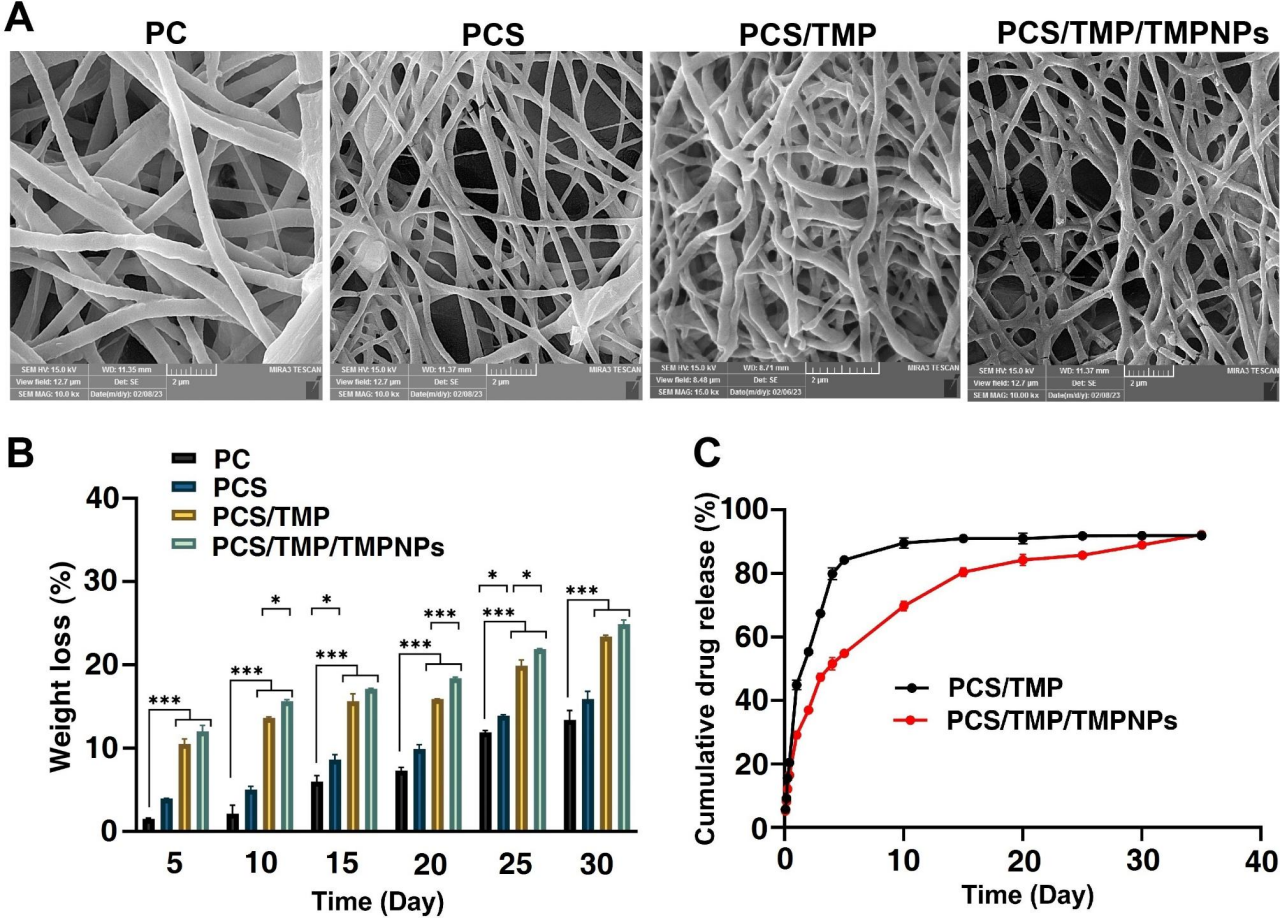
Characterization of the prepared nanofibrous mats in aqueous phase (**A-C**). Surface morphology of PC, PCS, PCS/TMP, and PCS/TMP/TMPNPs nanofiber degradation after 30 days soaking in DMEM in vitro (**A**). In vitro degradation of the prepared PC, PCS, PCS/TMP, and PCS/TMP/TMPNPs nanofibers at certain intervals of time (**B**). In vitro TMP release pattern in PBS at specific intervals of time (**C**). One-Way ANOVA with Turkey analysis (n = 3; *p < 0.05 and ***p < 0.001)

Polycaprolactone and polyethylene glycol are FDA-approved biodegradable polymers that are recently being investigated matrices for tissue-engineered applications [35]. Although PCL-based implants have been recognized for their biocompatibility, good mechanical properties, and low immunogenicity, undergo slow degradation, staying intact for up to 2 years in in vivo conditions [36]. PU-based nanofibrous mats have a structural resemblance to polyesters and polyamides, so their degradability is similar to that of polyesters and polyamides [37]. On the other hand, CS undergoes relatively fast degradation [38]. Based on previous results, the incorporation of CS in the scaffold increases significantly water uptake and weight loss [39]. As the optimal biodegradation rate of implants has a critical role in tissue engineering, the presence of CS would adjust the degradation rate of nanofibrous mats. Figure 4 A presents FE-SEM micrographs of nanofibrous PC, PCS, PCS/TMP, and PCS/TMP/TMPS degradation after 30 days of soaking in DMEM medium in in vitro conditions. A slight degradation rate and fewer cracks in the PC mats are visible in the structure of the nanofibers that which could be related to better mechanical stability during in vitro analyses. In contrast, the incorporation of CS promoted the hydrophilicity of PCS nanofibers, leading to deep fissures, mainly at junctions between fibers. Additionally, the FE-SEM images of PCS/TMP and PCS/TMP/TMPS exhibited that the surface of nanofibers becomes rough with a uniform degradation process after 30 days. Figure 4B represented the weight loss of the prepared nanofibers for various periods. The PC nanofibrous mat displayed a slow degradation rate, whereas the rate of weight loss of PCS nanofiber was higher than PC mat (*p* < 0.05). TMP blending with PCS leads to a higher degradation rate, and there were significant differences between PC and both PCS/TMP and PCS/TMP/TMPNPs nanofibrous mat degradation behaviors (*p* < 0.05).

## In vitro release tests

Figure 4 C indicates the in vitro TMP release behavior from both PCS/TMP and PCS/TMP/TMPNPs nanofibrous mats at 37˚C inside the aqueous medium with a pH value of 7.4. The standard curve of porphyrin was described in equation Y = 0.186X-0.334 with a correlation coefficient of 0.905, where X and Y represent the concentration (ppm) and the absorbance of TMP, respectively. The release kinetics of TMP from PCS/TMP and PCS/TMP/TMPNPs nanofibers was most rapid in the initial 24 h in which about 44.99 ± 1.46% and 29.19 ± 1.05% of the loaded TMP was released, respectively. Release profile analysis indicated that the use of CSNPs for loading TMP into nanofibers resulted in a more stable TMP release profile compared to PCS/TMP nanofibers over a long period. Of note, approximately 69.80 ± 1.539% of the loaded TMP was discharged within 10 days with a decreased initial burst release, while 89.66 ± 1.66% of TMP was released from PCS/TMP nanofibers during the same period. In PCS/TMP/TMPNPs nanofibers, as a result of the combination of initial rapid release and last prolonged release nearly 89.03 ± 0.82% of loaded TMP was released during 4 weeks with an initial rapid release of almost 54.83 ± 0.29% in the first 5 days.

### In vitro bactericidal properties

**Fig. 5:**
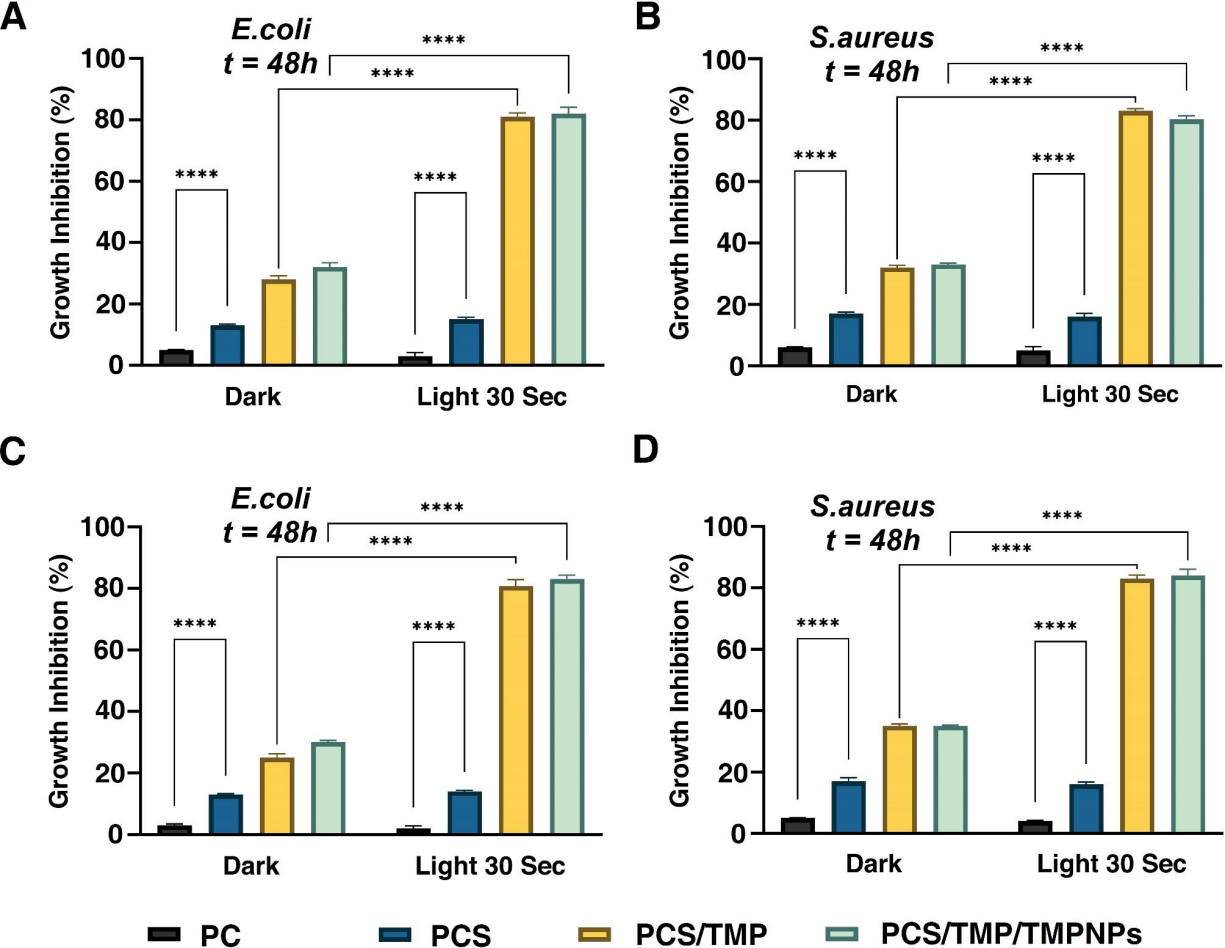
Antimicrobial properties of the fabricated nanofibrous mats (**A-D**). Antibacterial activities of the fabricated PC, PCS, PCS/TMP, and PCS/TMP/TMPNPs nanofibrous mats were assessed against *Escherichia Coli and Staphylococcus aureus* using turbidity (**A** and **B**), and MTT assays (**C** and **D**) in vitro after being exposed to 635 nm laser irradiation. The data are represented as mean ± SD. One-Way ANOVA with Turkey post hoc analysis (n = 3; ****p < 0.0001)

The presence of bacteria and other infectious agents at the site of injury can stimulate immune responses, and inflammation and delay the healing process [40]. To evaluate the antibacterial activities of the fabricated nanofibrous PC, PCS, PCS/TMP, and PCS/TMP/TMPNPs mats, both gram-negative *E.coli* and gram-positive *S.aureus* were selected as the leading bacteria pathogens. We noted that both nanofibrous PCS/TMP and PCS/TMP/TMPNPs mats exhibited proper antibacterial activity against *E*. *Coli* and *S. aureus* after being exposed to red laser light irradiation (Fig. 5 panels **A** and **B;***p* < 0.001). Several pieces of evidence point to the fact that photosensitizers can produce reactive oxygen species upon irradiation, leading to living cell damage and the inactivation of pathogenic microorganisms [41]. Because cutaneous tissue is in direct contact with diverse microbial agents, therefore, it seems that an ideal nanofibrous mat should be equipped with antimicrobial mechanisms to prohibit infection at the site of transplantation [42]. It is generally accepted that positively charged photosensitizers like cationic porphyrins can blunt both gram-negative and gram-positive bacteria [43]. Compared to negatively charged or neutral photosensitizers, cationic porphyrins are more effective against gram-negative strains [27]. The positive charge of me so-substituted cationic porphyrins leads to strong electrostatic interaction with negative components (lipopolysaccharide) in gram-negative bacteria [44]. Likewise, cationic porphyrins can inactivate *S. aureus* through electrostatic interactions with the negatively charged lipoteichoic acid on the bacterial surface. We also indicated that nanofibrous mats containing porphyrin exhibited less antibacterial properties in the dark place compared to the irradiated microenvironment. According to Fig. 5 panels **C** and **D**, similar results were observed using the MTT assay.

### In vitro assays

#### Hemolysis

**Fig. 6:**
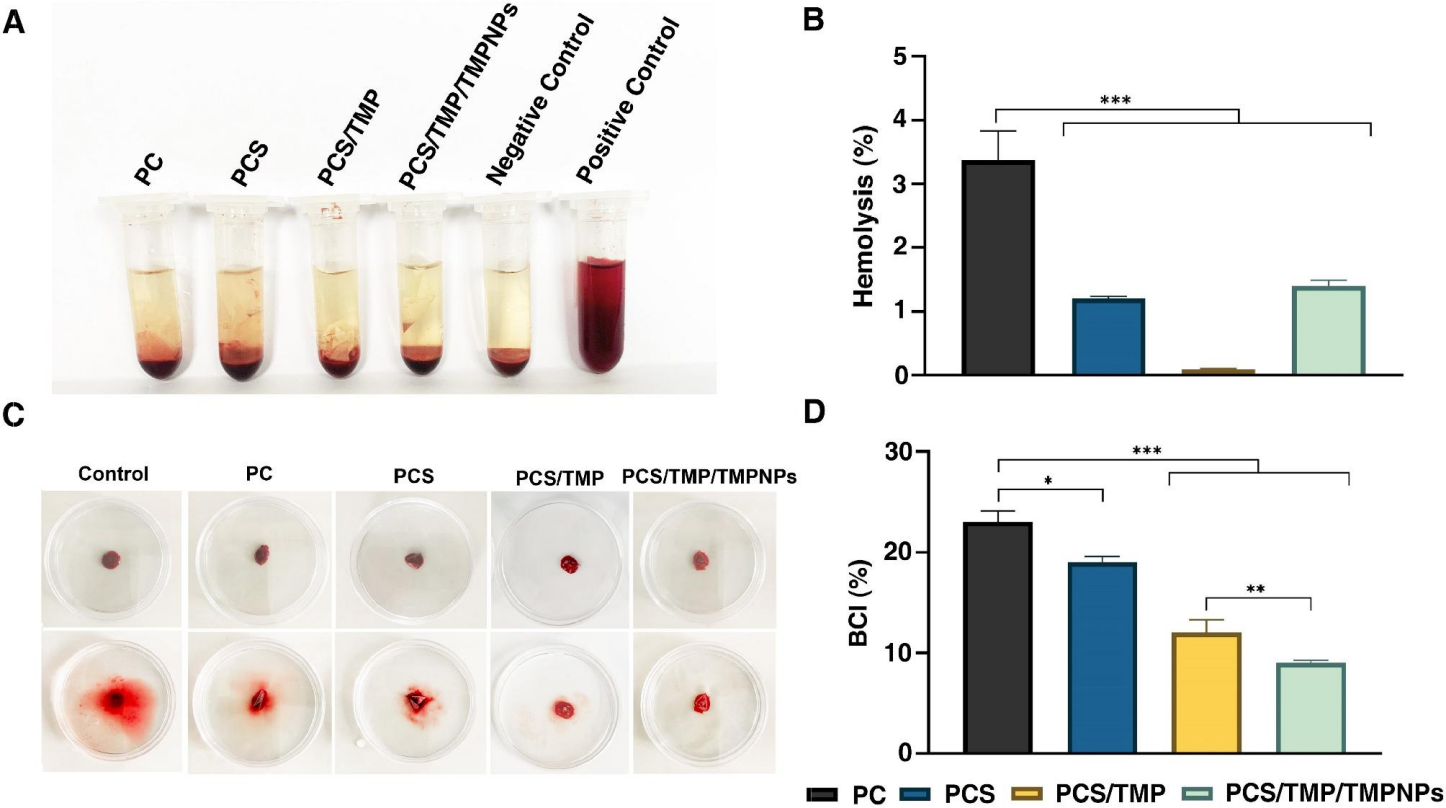
Hemolytic and blood clotting properties of the fabricated nanofibrous mats (**A-D**). Hemolytic activities of different groups of nanofibrous mats, blood in pure water were considered as the positive control, and blood solution in normal saline was considered as the negative control (**A**). Hemolysis percentage after incubation with red blood cells for 60 min at 37 °C (**B**). The image of the clot of developed nanofibrous mats before and after lysing (**B**) and BCI percentage of the prepared materials (**D**). Blood with pure water was chosen as a control group. One-Way ANOVA with Turkey analysis (n = 3; *p < 0.05, **p < 0.01, ***p < 0.001, and ****p < 0.0001)

The main indicator to assess the biocompatibility of nanofibrous mats is the rate of hemolysis [45]. Based on international standards, blood-biocompatible materials should possess a hemolytic value of less than 5% [46]. Figure 6 A shows the hemolysis ratio of the developed nanofibrous mats. The results revealed the maximum toxic nature of PC nanofibrous mats compared to the other groups (*p*_PC_ vs. _other groups_<0.001). The hemolytic ratio reached 3.37 ± 0.46% in the PC group while these values were 1.2 ± 0.03, 0.09 ± 0.01, and 1.43 ± 0.08 in PCS, PCS/TMP, and PCS/TMP/TMPNPs groups. As indicated in Fig. 6B, the hemolysis ratio for all groups was less than 4%, indicating good hemo-biocompatibility.

#### Blood clotting index

BCI test and rapid formation of a clot are appropriate in vitro assays to examine the blood clotting capability of fabricated scaffolds. This test is based on the activation of intrinsic coagulation cascades and indicated trapped RBCs within the fibrin clots [47]. Therefore, less hemoglobin value represents favorable hemostatic performance. PCS/TMP/TMPNPs nanofibrous mats exhibited visibly lighter color and consequently the best BCI performance (Fig. 6C). It seems that the positive charge of TMP was the major reason for the low BCI value. In this study, the BCI value for the PC nanofibrous mat reached 23 ± 1.12%, indicating a weak function of this sample in forming a stable clot. The results indicated that the BCI values for developed nanofibrous PCS, PCS/TMP, and PCS/TMP/TMPNPs mats were 19 ± 0.58, 12 ± 1.30, and 9 ± 0.23%, respectively (Fig. 6D; *p*_PC_ vs. _PCs_ <0.05; *p*_PC_ vs. _PCS/TMP, and PCS/TMP/TMPNPs_ <0.001). According to our data, the minimum BCL index was achieved in PCS/TMP/TMPNPs group in which we found statistically significant differences compared to PCS/TMP group (*p*_PCS/TMP_ vs. _PCS/TMP/TMPNPs_ <0.01). Guo et al. prepared different types of 3D loose nanofiber sponges based on polyurethane and measured the blood clotting index to evaluate the hemostatic ability of the sponges. It was reported that PU- tranexamic acid (TA)/gelatin sponge indicated significantly lower BCI values (30.42 ± 0.91%) compared to other sponges [48]. In another study, Liu et al. fabricated polyurethane-urea foam (PUUF) wound dressing via the particle leaching and freeze-drying technique. The PUUF wound dressing showed a better blood coagulation effect than Gauze and commercial polyurethane dressing (CaduMedi). In this study, the BCI value of Gauze was 83.4%, in 10 min, which was the biggest of than other samples. The BCI of CaduMedi was 50.6% at 10 min, while the BCI of PUUF reaches 21.9% [49].

## Proliferation assay and cell adhesion assay

**Fig. 7:**
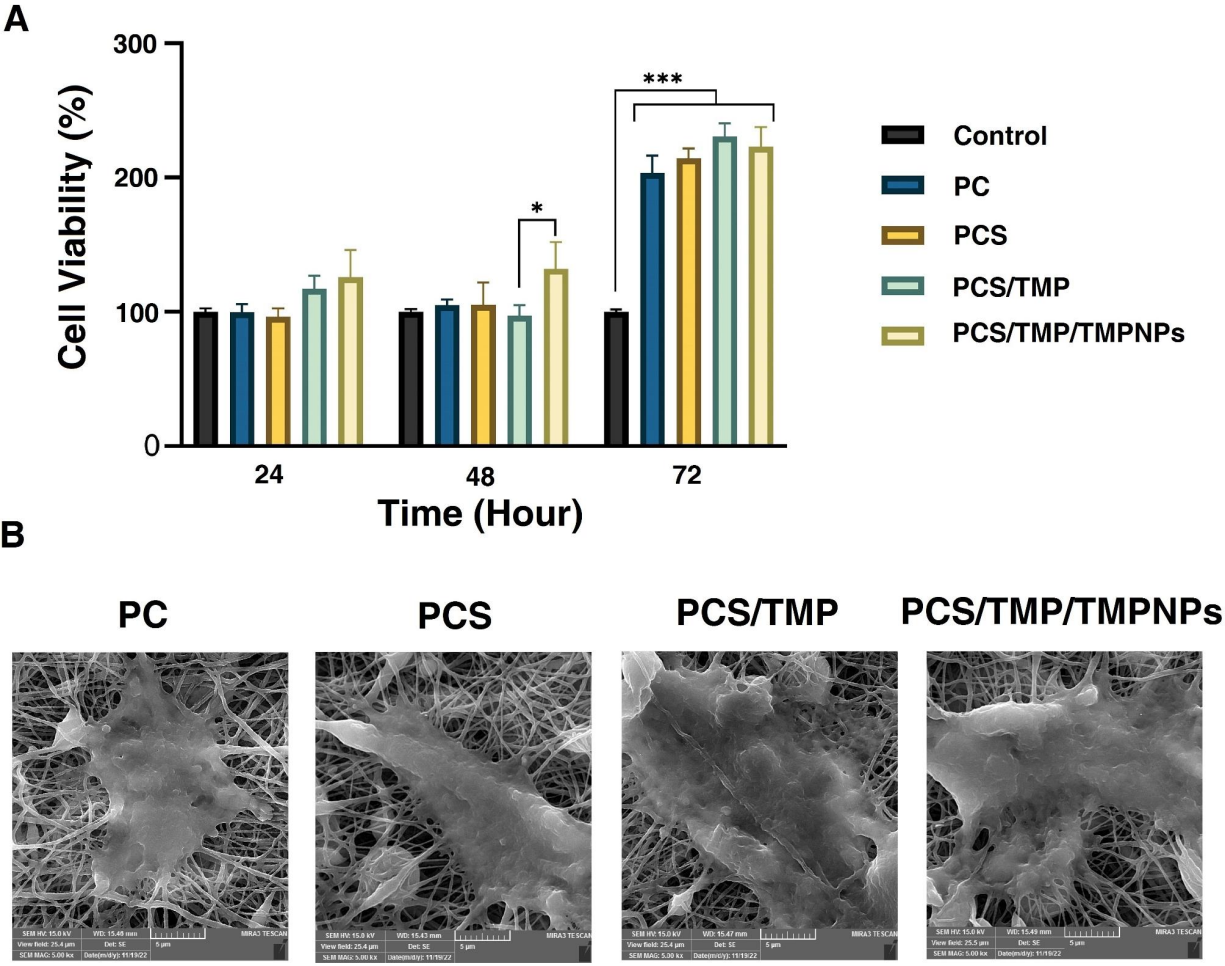
Measuring the viability of HFFF2 cells after being plated on the surface of PC, PCS, and PCS/TMP/TMPNPs over 72 h. In vitro biocompatibility of HFFF2 fibroblasts was examined using an MTT assay (**A**). Data indicated a statistically significant increase in survival rate in PC, PCs, PCS/TMP/TMPNPs groups compared to the plastic surface-coated cells (p < 0.05). FE-SEM micrographs of HFFF2 cells seeded on PC, PCS, PCS/TMP, and PCS/TMP/TMPNPs nanofibrous mats indicated flattened morphology with several projections after the periphery of each cell (**B**). The data are presented as mean ± SD. One-Way ANOVA with Turkey analysis (n = 3; *p < 0.05 and ***p < 0.001)

In the present study, the effect of designed nanofibrous mats was examined on human HFFF2 fibroblasts using an MTT assay after 24, 48, and 72 h. The results showed the culture of HFFF2 fibroblasts on all nanofibrous mats increased the survival rate after 72 h compared to the cells plated on the plastic surface (Fig. 7A; p < 0.05). While the statistically significant difference was found in terms of survival rate after 48 h in PCS/TMP/TMPNPs compared to the control group (p < 0.05). We found non-significant differences between the nanofibrous mats in all time points (p > 0.05). Based on the data, PCS/TMP/TMPNPs exert the maximum effects on HFFF2 fibroblast viability.

The morphology of plated HFFF2 fibroblasts was studied on nanofibrous mats after 72 h (Fig. 7B). Data showed can attach appropriately on the surface of PC, PCS, PCS/TMP, and PCS/TMP/TMPNPs and acquired flattened morphologies. After plating on the nanofibrous surface, HFFF2 fibroblasts extended projections in different directions, indicating an appropriate interaction between the cells and nanofibrous mats. These findings revealed that the developed mats are the appropriate platforms for the attachment and morphological adaptation of fibroblasts.

### In vivo studies

#### Wound closure rate

**Fig. 8:**
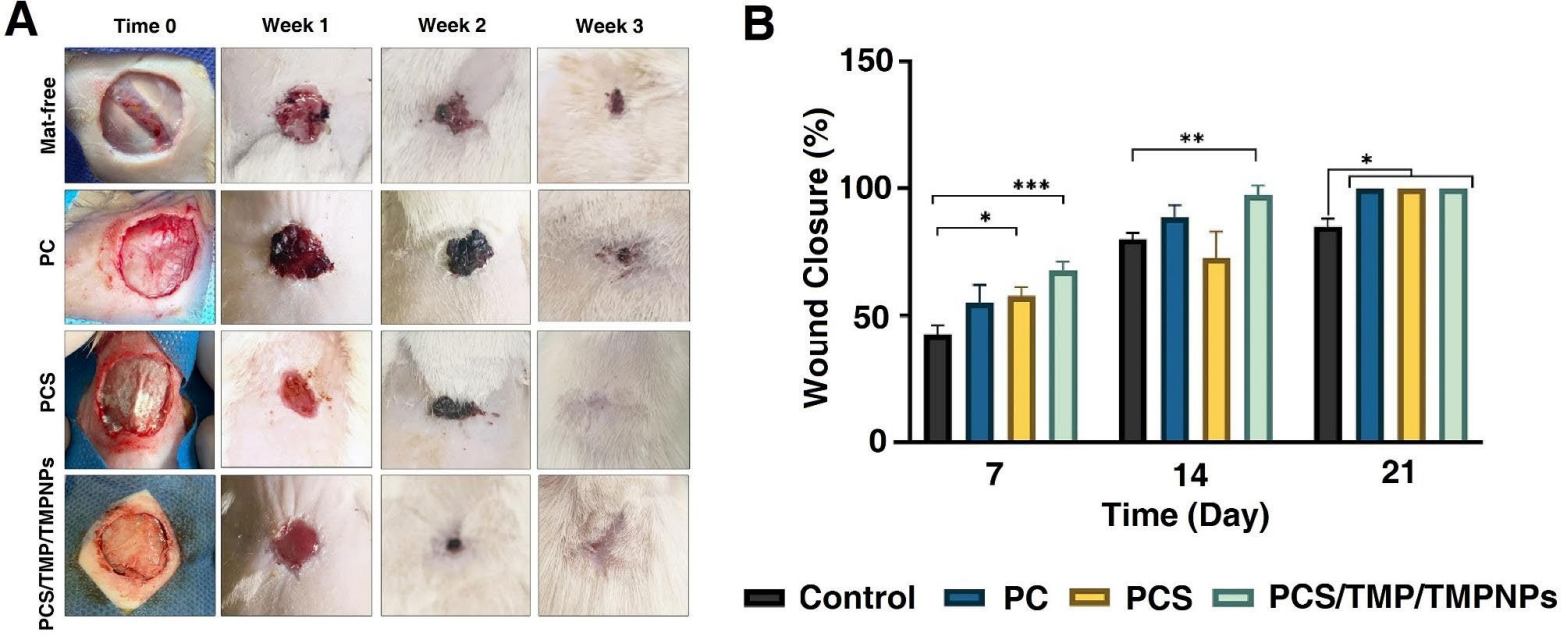
Wound healing properties of PC, PCS, and PCS/TMP/TMPNPs in a rat model of full-thickness excisional wound. Photographs of wound closure percentage in different groups at weeks 1, 2, and 3 after implantation (**A**). Data indicate that the wound closure rate was faster and higher in the groups that received nanofibrous mats compared to the mat-free rats (p < 0.05). We found non-significant differences in wound closure percent between the PC, PCS, and PCS/TMP/TMPNPs at all-time points (p > 0.05). The wound closure rate and contraction ability were more prominent in the PCS and PCS/TMP/TMPNPs rats compared to the other groups (**B).** The data are presented as mean ± SD. One-Way ANOVA with Turkey analysis (n = 3; *p < 0.05, **p < 0.01, and ***p < 0.001)

**Fig. 9:**
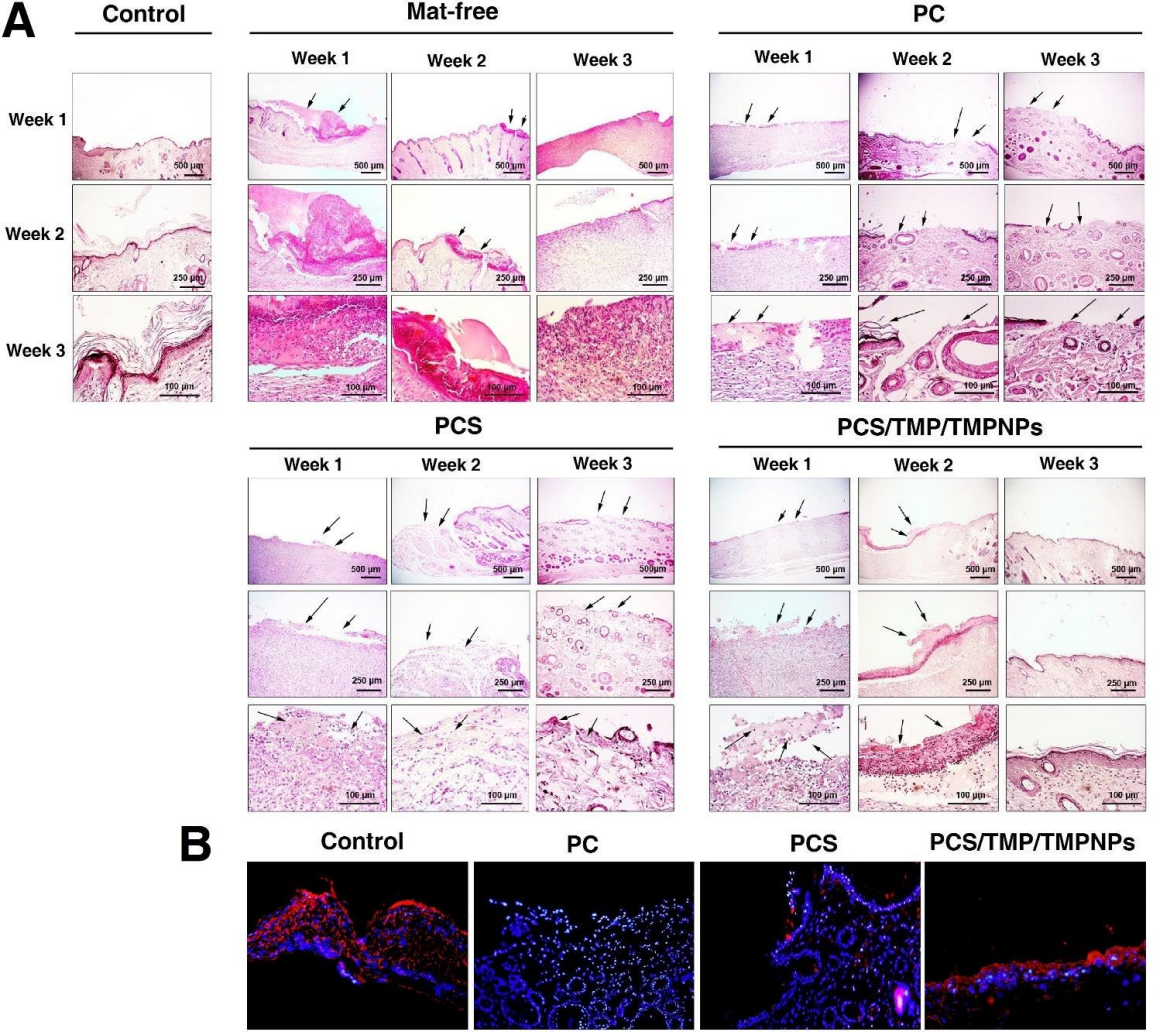
Histological analysis of skin biopsies. H & E staining revealed the existence of significant inflammatory cells and exudate (black arrows) in the mat-free rats compared to the other groups (**A**). The application of electrospun PC, PCS, PCS/TMP/TMPNPs can reduce the excessive inflammatory response, exudate, and fibrotic changes. These properties were more prominent in the PCS/TMP/TMPNPs group. Unlike PC and PCS groups, placing PCS/TMP/TMPNPs mats at the site of injury can lead to the restoration of epidermis structure after three weeks. Monitoring the Desmoglein protein levels in different groups using IF images (**B**. Magnification 20X). Data showed that PCS/TMP/TMPNPs efficiently promote the synthesis of Desmoglein compared to other experimental groups, resulting in appropriate cell-to-cell connection

We examined the healing potential of cutaneous tissue in a rat model of the full-thickness excisional wound during the three weeks (Fig. 8A). Based on obtained data, the wound closure rate was higher and faster in groups that received topically PC, PCS, PCS/TMP/TMPNPs mats compared to the mat-free rats (*p* < 0.05; Fig. 8B). The rate of wound closure increased over time and reached maximum levels after three weeks. We found non-significant differences in terms of wound closure rate between the PC, PCS, and PCS/TMP/TMPNPs. Based on the data; the contraction ability at the site of injury was more prominent in PCS and PCS/TMP/TMPNPs groups. Bright-field imaging exhibited massive exudate with significant inflammatory cell infiltration at the site of injury in mat-free rats (black arrows) (Fig. 9A). We found that the volume and intensity of exudate and inflammatory cells were diminished after two weeks (black arrows). On week 3, acute inflammatory responses were relatively switched off accompanied by the formation of fibrotic changes and non-aligned fibroblast at the site of injury. In the group that received PC, the full-thickness excisional area had less inflammatory response compared to the nanofibrous mats-free rats at the respective similar time points (black arrows). Despite the lack of prominent exudate and inflammatory cells, a full-thickness excisional area can be easily detected after three weeks. In the PCS group, the intensity and volume of exudate were less compared to the mat-free and PC rats (black arrows). The density of fibroblasts was also less in the PCS rats compared to the full-thickness excisional area without the implantation of nanofibrous mats. Despite data from PC and PCS groups, the remnant of nanofibrous mats can be readily indicated in the group that received PCS/TMP/TMPNPs (black arrows) containing vascular units and inflammatory cells. On week 2, the mat line placed on the excisional area is detectable. Of note, we found that the full-thickness excisional area was filled with the epithelial cell layer without any inflammatory response and fibrotic changes. These features indicate that topical implantation of PCS/TMP/TMPNPs at the site of full-thickness excisional areas restores the histological properties of cutaneous tissue after three weeks. Using immunofluorescence assay, we monitored protein levels of Desmoglein and cutaneous tissue integrity (Fig. 9B). Data indicated a lack of Desmoglein in the PC group compared to control healthy cutaneous counterparts and this factor is barely detectable in rats that received PCS. We note the increase of Desmoglein in the PCS/TMP/TMPNP group compared to PCS and PC, indicating the promotion of cell-to-cell communication and physical contact in the presence of PCS/TMP/TMPNP electrospun mat.

## Conclusion

This study aimed to assess the healing potential of PCS/TMP/TMPNPs on wound healing in rats. Here, it was shown that placing PCS/TMP/TMPNPs in the target injury site in cutaneous tissue can accelerate the healing rate with the reduction of fibrotic changes, bacterial infection, and promotion of epithelialization. The existence of photosensitive porphyrin prohibits unwanted infection with gram-positive and gram-negative bacteria which seems more critical in microenvironments in close contact with commensal infectious agents. The current study faces some limitations that need further consideration. In this study, we used HFFF2 cells for the evaluation of the cytocompatibility assay. The selection of other cell types from epithelial compartments, especially epidermal keratinocytes, can give us invaluable data about the regenerative potential of PCS/TMP/TMPNPs after skin injuries. It is suggested future studies focus on the evaluation of other markers related to dermal wound healing. Monitoring specific immune responses is mandatory to predict the status of healing, and dominant cell types at the site of transplantation. Future studies can examine the possibility of this composite for keratinized and non-keratinized cutaneous layers. Taken together, this study highlights the importance of certain electrospun mats with specific physiochemical properties in the regeneration of cutaneous tissue. It is suggested that transplanted scaffolds should possess specific physicochemical characteristics for skin injuries compared to those used for other tissues. Besides, the importance of mechanical properties, cytocompatibility, and biological features, the application of photosensitizers as ROS donors can prohibit subsequent bacterial infections. Future studies should focus on the fabrication of different composites with anti-bacterial agents for skin tissue engineering purposes.

## Data Availability

Data will be available on reasonable request.

## Abbreviations

APDT: Antimicrobial photodynamic therapy
CS: Chitosan
CSNPs: CS tripolyphosphate nanoparticles
DMSO: Dimethyl sulfoxide
DMEM/HG: Dulbecco’s Modified Eagle Medium
E. coli: Escherichia coli
FBS: Fetal Bovine Serum
IF: Immunofluorescence
TMP: Meso tetrakis (N-methyl pyridinium-4-yl) porphyrin tetratosylate salt
DMF: N, N-dimethylformamide
NPs: Nanoparticles
Pen-Strep: Penicillin-Streptomycin
PBS: Phosphate-buffered saline
PCEC: Poly (caprolactone)-Poly (ethylene glycol)–poly (caprolactone)
PU: Polyurethane
PC: PU/PCEC
PCS: PU/PCEC/CS
ROS: Reactive oxygen species
SEM: Scanning electron microscope
Sn(Oct)_2_: Stannous 2-ethyl hexanoate
WCA: Water contact angle
